# Pharmacokinetics of acute sertraline overdose: an observational retrospective study

**DOI:** 10.3389/fphar.2026.1781140

**Published:** 2026-03-30

**Authors:** Renzhu Liu, Qichang Xing, Zheng Liu, Wencan Li, Keqian Chen, Haibo Lei, Xiang Liu

**Affiliations:** 1 Department of Clinical Pharmacy, The Central Hospital of Xiangtan (The Affiliated Hospital of Hunan University), Xiangtan, China; 2 School of Biomedical Sciences, Hunan University, Changsha, Hunan, China

**Keywords:** overdose, pharmacokinetics, poisoning management, sertraline, therapeutic drug monitoring

## Abstract

**Background:**

Sertraline, a selective serotonin reuptake inhibitor is commonly prescribed for adolescent patients with depression, either as monotherapy or in combination with other treatments. Therapeutic doses typically range from 50 to 300 mg/day, whereas overdose cases generally exceed 450 mg, as observed in this study. This study aimed to characterise the pharmacokinetic profiles of sertraline in acute overdose settings and evaluate the impact of gastric lavage on drug clearance.

**Methods:**

This observational case retrospective study analysed the clinical manifestations, severity, treatment interventions, and outcomes of sertraline overdose cases.

**Results:**

The dataset comprised 16 cases of sertraline overdose. Gastric lavage was administered between 0.67 h and 12 h post-ingestion, with blood sampling commencing after lavage completion. The median ingested dose was 925 mg (range: 450–1,400 mg). Apparent elimination half-lives, estimated from two-point concentration measurements obtained 1–22 h and 27–146 h post-ingestion, ranged from 13.27 to 52.78 h (median: 24.12 h). These estimates represent post-decontamination kinetics and may not reflect true elimination half-lives due to unknown peak concentration timing. No life-threatening toxicity or severe organ dysfunction was observed.

**Conclusion:**

This study characterized post-gastric lavage sertraline concentrations in 16 adolescent intentional overdose cases. Apparent half-life estimates overlapped with therapeutic ranges, but methodological constraints, including unknown Tmax and two-point sampling, preclude definitive conclusions regarding overdose pharmacokinetics. Gastric lavage appeared well-tolerated and may reduce drug exposure, though its impact on clinical outcomes requires further study. These preliminary observations require validation in prospective studies with serial sampling.

## 1 Introduction

Suicide is the second leading cause of death among adolescents aged 10–19 years (Mangione et al., 2022). Recent reports indicate that, in 2023, approximately 40% of high school students experienced persistent sadness or hopelessness, 18% were diagnosed with major depression, and 10% reported having attempted suicide (Woolf, 2025). Recent research suggests a concerning prevalence of depression among United States (US) adolescents aged 13–18 years, with 15%–20% of this population affected (Miller and Campo, 2021). Notably, a substantial proportion of these individuals also experience co-occurring substance use disorders, highlighting a critical intersection of mental health and behavioural challenges in this age group. Depression in adolescents is commonly accompanied by changes in sleep patterns, appetite or body weight, inattention or lack of energy, and recurrent thoughts of death or suicide. Selective serotonin reuptake inhibitors (SSRIs) and serotonin-norepinephrine reuptake inhibitors (SNRIs) are commonly prescribed for adolescent depression, with sertraline (SER) being the most frequently used SSRI (Molero et al., 2025). Studies demonstrate that SER, in combination with cognitive therapy or repetitive transcranial magnetic stimulation (rTMS), improves depressive symptoms in adolescents and maintains an acceptable safety profile. Ingestion of large drug doses is a recognised method of suicide among patients with depression (Shu et al., 2025).

SER, chemically designated as (1S,4S)-4-(3,4-dichlorophenyl)-1,2,3,4-tetrahydro-N-methyl-1-naphthalenamine, is a naphthylamine derivative (Arabkhani et al., 2022). Its primary metabolite, N-desmethylSER (DSER), is produced through extensive first-pass metabolism via N-demethylation after oral administration (Drugbank, 2025). DSER has low affinity for the serotonin receptor and is not associated with substantial clinical effects (Doherty et al., 2007).

While therapeutic dosing for depression typically ranges from 50 to 300 mg/day, doses may be escalated to 400–2000 mg/day in cases of acute depressive episodes, depending on clinical response and tolerability. In this study, intentional overdose cases involved doses exceeding 450 mg. In this study, intentional overdose cases involved doses exceeding 450 mg. Following metabolism, the parent drug content is relatively low. Analytical methods reported in the literature generally include liquid chromatography–tandem mass spectrometry (LC-MS/MS) (Wang et al., 2024; Phogole et al., 2023; Fariha et al., 2023), or gas chromatography–tandem mass spectrometry (GC-MS/MS) (Cabarcos-Fernandez et al., 2021; Luiz et al., 2020), with some studies using high-performance liquid chromatography with ultraviolet detection (HPLC-UV) (Chaves et al., 2010). Mass spectrometry (MS) offers high sensitivity, but is costly and requires specialised maintenance, limiting its accessibility in routine clinical laboratories. Ultraviolet detectors are more cost-effective, providing sufficient sensitivity to meet the required limits of the detection (LOD) and quantification (LOQ). In this study, large-volume injection was applied after protein precipitation. Preliminary separation was performed on a one-dimensional chromatographic column, and analytes corresponding to the target retention times were collected for subsequent analysis on a two-dimensional chromatographic column. This approach ensured accurate, rapid analytical results, providing a foundation for clinical adjustments in the management of SER poisoning. This study aimed to characterise the pharmacokinetic profiles of sertraline in acute overdose and to evaluate the impact of gastric lavage on drug clearance, which is critical for optimising treatment timing and improving clinical outcomes.

## 2 Methods

### 2.1 Inclusion and exclusion criteria

Medical records of patients with acute SER overdose poisoning treated at Xiangtan Central Hospital from January 2022 to October 2025 were retrospectively reviewed. Consecutive cases of acute SER overdose in adolescents aged 12–20 years were included. Patients with incomplete medical records or whose presentation was unrelated to overdose were excluded. Acute overdose was defined as a single ingestion exceeding 450 mg, which is above the maximum recommended therapeutic dose of 300 mg/day.

### 2.2 Development and validation of SER serum drug concentration methodology

Protein precipitation is a widely used and efficient method for sample purification. It is simple to operate, requires minimal reagent volumes, and reduces the potential for human error. Methanol or acetonitrile is added to the sample, thoroughly mixed, and then centrifuged. The resulting supernatant is then injected into a chromatographic column for analysis. This technique can serve as an independent purification step (Zhang et al., 2011; Domingues et al., 2016).

The instruments used included a Shimadzu LC-20AT high-performance liquid chromatograph comprising an SIL-20A automatic sampler, three LC-20AT pumps, an SPD-20A ultraviolet detector, a DGU-20A online degasser, and a CBM-20A system controller (Shimadzu Corporation, Kyoto, Japan), in addition to a coupling instrument FLC-2801 (Hunan Demeter Instrument, Changsha, China). Chromatographic separation was performed using a two-dimensional column Aston SCB (125 mm × 4.6 mm, 5 μm), a one-dimensional column SX1 (25 mm × 3.5 mm, 5 μm), and an intermediate column SCB (10 mm × 4.6 mm, 3.5 μm). The two-dimensional mobile phase consisted of API3-2A and MPI-2A; the one-dimensional mobile phase was CAA-1, and the auxiliary mobile phase was double-distilled water. The flow rate was 1.2 mL/min, the detection wavelength was 225 nm, the injection volume was 500 μL, and the column temperature was maintained at 40 °C. Plasma concentrations of SER in patients with overdose were determined using 2D-HPLC. Sixteen patients received toxic doses of SER, ranging from 450 to 1,400 mg. Venous blood (3 mL) was collected from each patient into an ethylenediaminetetraacetic acid (EDTA) vacuum tube. Plasma was separated by centrifugation at 3,000 rpm (1,260 × g) for 5 min and subsequently analysed.

### 2.3 Data collection

Demographic characteristics, clinical features, and laboratory test results were collected for each patient. Symptom severity at admission was systematically evaluated using the Hunter Serotonin Toxicity Criteria. The clinical course, including treatment regimens and outcomes, was recorded.

### 2.4 Treatment measures

All patients with SER poisoning received gastric lavage upon admission to remove unabsorbed drugs. Blood samples were collected post-lavage to measure SER concentrations. Additional symptomatic treatments included proton pump inhibitors to protect the gastrointestinal tract, vitamin B6, lactulose, and fluid supplementation to facilitate drug elimination. Follow-up SER measurements were performed to guide further treatment.

### 2.5 Ethical considerations

The study was conducted in accordance with the principles of the Declaration of Helsinki. The protocol was approved by the Institutional Ethics Committee of Xiangtan Central Hospital (Approval No. 2022-05-005). As this was a retrospective analysis of anonymised medical records, the requirement for written informed consent was waived. Patient anonymity and data confidentiality were strictly maintained. The study adhered to the STROBE guidelines for observational studies.

### 2.6 Statistical analysis

Descriptive statistical methods were used in accordance with observational non-comparative study design requirements. Continuous variables, including age, laboratory parameters, and length of hospital stay, were non-normally distributed and therefore reported as median (range).

Categorical variables, such as sex, concomitant medications, complications, and treatment regimens, were presented as frequency and percentage. Non-parametric tests were used to explore potential associations between clinical variables and drug clearance. Mann-Whitney U tests were used to compare continuous variables between two groups, whereas Kruskal–Wallis H tests were used for comparisons across multiple groups. Post-hoc pairwise comparisons were adjusted using the Bonferroni correction. Correlation coefficients (Pearson’s r) were calculated for continuous variables. All tests were two-tailed with a significance threshold of α = 0.05. Statistical analyses and data tabulation were performed using Python 3.13 software, and the results were independently reviewed by two researchers to ensure accuracy.

## 3 Results

### 3.1 Basic characteristics of patients with SER poisoning

A total of 16 patients were included in this study, comprising 3 males (18.75%) and 13 females (81.25%). The median age was 15 years (range: 12–20 years). All patients had experienced acute SER poisoning, with a median ingested dose of 925 mg (range: 450–1,400 mg). Some patients had concurrently used other medications, including quetiapine, trazodone, and valproic acid. Demographic and clinical information is summarised in Table 1.

**TABLE 1:** Demographic information of the patients with overdose SER.

Characteristics	Median (range)
Gender (N,%)	
Male	3 (18.75%)
Female	13 (81.25%)
Age (year)	15 (12-20)
Body weight (kg)	38 (33-42)
Depression	16 (100%)
Dose of SER (mg)	925 (450-1,400)
Combined drugs (N)	
Quetiapine	4
Trazodone	3
Valproic acid	1
Mirtazapine	1
Risperidone	1
Lithium carbonate	1
Benzodiazepines	1
Bifidobacterium	1

The principal clinical manifestations involved the nervous, gastrointestinal, and cardiovascular systems. Serotonin syndrome was diagnosed in all patients (100%) according to the Hunter criteria, with seven cases presenting severe manifestations that required intensive care. Details are provided in Table 2.

**TABLE 2:** Clinical characteristics of patients with overdose SER.

Id	Clinical symptoms	Hunter serotonin toxicity criteria
1	Drowsiness (altered mental status), vomiting (gastrointestinal), white vomitus (possible hypersecretion)	3
2	Nasal congestion (autonomic), diarrhea (gastrointestinal), abdominal pain (visceral), fatigue (generalized)	4
3	Fever (temperature >38 °C), shivering (autonomic), body aches (generalized)	3
4	Dizziness (neurological), hyperthermia (autonomic), tremor (neuromuscular)	3
5	Hand tremor (neuromuscular), dizziness (neurological), nausea (gastrointestinal)	3
6	Dizziness (neurological), upper abdominal pain (visceral), vomiting (gastrointestinal), confusion (altered mental status)	4
7	Nausea (gastrointestinal), vomiting (gastrointestinal), chest tightness (cardiovascular), dyspnea (respiratory)	5
8	Vomiting (gastrointestinal), abdominal pain (visceral), dizziness (neurological), malaise (generalized)	4
9	Nausea (gastrointestinal), palpitations (cardiovascular), ataxia (neurological), dyspnea (respiratory)	5
10	Palpitations (cardiovascular), tachycardia (autonomic), anxiety (behavioral)	3
11	Chest distress (cardiovascular), hyperventilation (respiratory), diaphoresis (autonomic)	3
12	Vomiting (gastrointestinal), abdominal pain (visceral), dizziness (neurological), headache (neurological)	4
13	Headache (neurological), tremor (neuromuscular), photophobia (sensory), restlessness (behavioral)	4
14	Blurred vision (visual), mydriasis (ocular), dry mouth (autonomic)	3
15	Agitation (behavioral), shivering (autonomic), nausea (gastrointestinal), headache (neurological)	5
16	Flushing (autonomic), paresthesia (neurological), somnolence (altered mental status), tachypnea (respiratory)	4

### 3.2 Methodological validation of SER serum drug concentration monitoring

**FIGURE 1:**
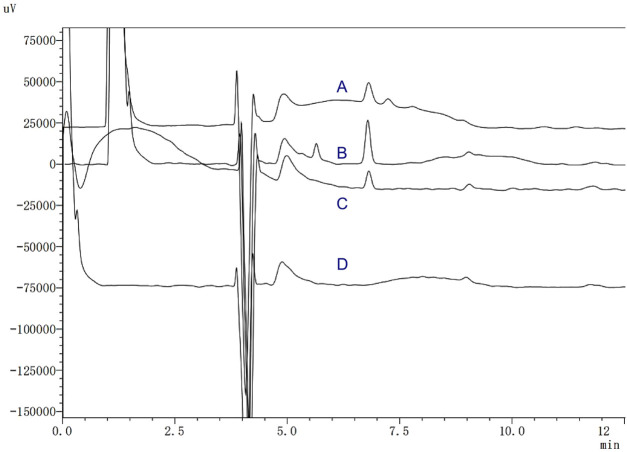
The typical chromatograms of **(A)** patient plasma with SER, **(B)** blank plasma spiked with SER, **(C)** SER substance solubilized by one-dimensional mobile phase and **(D)** blank plasma.

**FIGURE 2:**
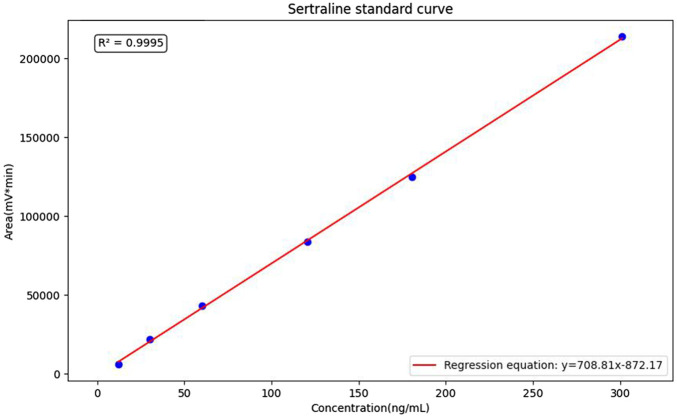
Sertraline standard curve.

Figure 1 illustrates representative chromatograms under four distinct conditions: blank plasma, SER solubilised in the one-dimensional mobile phase, blank plasma spiked with SER, and patient plasma containing SER. The retention time of SER was 12.5 min, enabling rapid clinical detection. Chromatographic peaks were well-defined, and detection signals were strong. Endogenous substances in blank plasma, human plasma, and mobile phase did not interfere with the analyte peak, indicating good selectivity and absence of significant matrix effects. Standard solutions were processed using the described sample preparation method, and peak areas of SER were measured. Linear regression was performed with peak area as the ordinate and analyte concentration as the abscissa. The regression equation for the SER standard curve was Y = 708.81X−872.17 (R2 = 0.9995, RSD% = 12.24). The calibration curve is shown in Figure 2.

### 3.3 Pharmacokinetic characteristics of patients

Pharmacokinetic parameters of SER are presented in Table 3.

**TABLE 3:** Pharmacokinetic characteristics of patients with overdose SER.

Id	Sertraline dosage	Pre-admission treatment measures	Time from drug poisoning to admission	Gastric lavage with normal saline	Treatment	The first monitoring of sertraline concentration time (t_1_)	First monitoring of sertraline concentration (ng/mL) (C_1_)	The second monitoring of sertraline concentration time (t_2_)	Second monitoring of sertraline concentration (ng/mL) (C_2_)	0utcome
1	50 mg*9	Vomiting	2 h	Yes	NaCl, PPI	4 h	298.38	58 h	146.8	Improved
2	50 mg*28	Vomiting	12 h	Yes	NaCl, VB6	12 h	621.95	52 h	200.37	Improved
3	50 mg*24	No	4 h	Yes	NaCl, lactulose oral solution	22 h	112.86	58 h	48.08	Improved
4	50 mg*28	No	1 h	Yes	NaCl, VB6, VC, oral rehydration salt	2 h	371.95	80 h	50.08	Improved
5	50 mg*16	No	1 h	Yes	NaCl, VB6	2 h	336.75	37 h	110.15	Improved
6	50 mg*12	No	1.5 h	Yes	NaCl, VB6	2 h	268.51	146 h	9.96	Improved
7	50 mg*14	No	7 h	Yes	NaCl, VB6	1 h	187.74	47 h	22.72	Improved
8	50 mg*20	No	1 h	Yes	NaCl, VB6	2 h	252.78	33 h	76.37	Improved
9	50 mg*20	No	4 h	Yes	NaCl, VB6	1.5 h	801.1	46 h	149.06	Improved
10	50 mg*14	No	5 h	Yes	NaCl, VC	1.5 h	415.98	32.5 h	197.99	Improved
11	50 mg*14	No	2 h	Yes	NaCl, VB6	2 h	338.97	44 h	89.61	Improved
12	50 mg*28	Vomiting 3 times	3 h	Yes	NaCl, VB6, VC	2 h	348.86	60 h	90.74	Improved
13	50 mg*17	No	2 h	Yes	NaCl, VB6	1 h	455.11	31 h	135.69	Improved
14	50 mg*28	No	3 h	Yes	NaCl, VC	2 h	298.77	27 h	80.98	Improved
15	50 mg*11	No	11 h	Yes	NaCl, VB6, PPI	1.5 h	242.06	47.5 h	50.36	Improved
16	50 mg*14	No	40 min	Yes	NaCl, sodium bicarbonate, lactulose oral solution, flumazenil	10 h	441.27	40 h	135.69	Improved

NaCl, Sodium chloride solution; PPI, Proton pump inhibitors; VB6,Vitamin B6; VC, Vitamin C.

A: Half-life calculated as t_1/2_= (t_2_-t_1_)* ln (2)/ln (C_1_/C_2_), assuming first-order elimination between two points.

B: All first samples were collected after gastric lavage; true peak concentration (Tmax) timing is unknown.

C: Apparent half-life estimates may reflect combined absorption-distribution-elimination phases.

**FIGURE 3:**
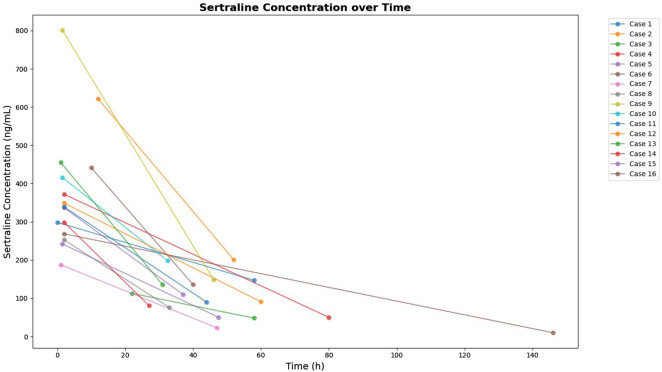
Sertraline concentration over time.

All patients underwent gastric lavage within 0.67–12 h post-ingestion. Fluid replacement was achieved using normal saline and oral rehydration solutions. Lactulose was administered to some patients to regulate osmotic pressure and enhance drug excretion. The mean half-life of SER clearance was 24.12 h (range: 13.27–52.78 h), representing the time required for plasma drug concentration to decrease by half. Owing to clinical constraints, continuous sampling was not feasible; hence, a two-point method was employed to estimate the half-life, with the first sample collected 1–12 h post-ingestion and the second at 46 ± 18 h. This method may underestimate the influence of the distribution phase, reflecting the elimination phase kinetics predominantly. Changes in SER plasma concentration are shown in Figure 3. Age exhibited a significant inverse correlation with half-life (r = −0.41, p = 0.032), with younger patients (<15 years) demonstrating prolonged clearance (median 28.98 h vs. 21.08 h). Co-administration of cytochrome P450 2D6 (CYP2D6) inhibitors extended the half-life by 45% compared with monotherapy (25.24 h vs. 13.26 h, p = 0.008). The CYP2D6 inhibitors identified in this study included quetiapine, trazodone, and risperidone, all of which have been reported to inhibit CYP2D6 activity to varying degrees. Vomiting before admission was associated with delayed clearance (34.24 h vs. 21.45 h, p = 0.002). These findings suggest potential pharmacokinetic interactions that warrant further validation. Detailed data are provided in Table 4.

**TABLE 4:** Non-parametric analysis of clearance factors.

Factor	Comparison groups	p-value	Effect size (r)
Age	<15 vs. ≥ 15 years	0.032*	−0.41
Gender	Male vs. female	0.185	−0.22
Co-administration	Alone vs. combination	0.008**	0.48
Vomiting	Present vs. absent	0.002***	0.53
CYP2D6 inhibition	Yes (quetiapine, trazodone, risperidone) vs. No	0.014*	0.43

**FIGURE 4:**
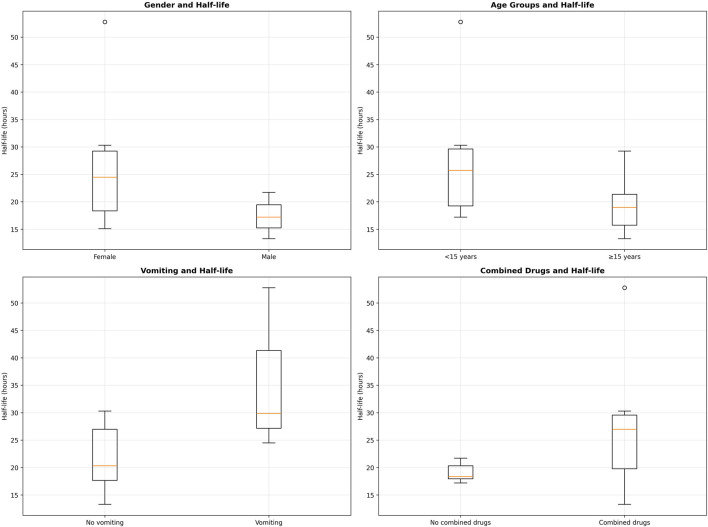
Factors affecting the elimination half-life of sertraline.

Concurrent use of other medications may result in drug–drug interactions that impede SER metabolic clearance. Pharmacokinetic profiles according to co-medication status are illustrated in Figure 4.

## 4 Discussion

Through an observational case series analysis of SER overdose cases reported at Xiangtan Central Hospital from 2022 to 2025, acute SER overdose was generally well-tolerated, and no life-threatening events were observed.

### 4.1 Excessive and severe cases of SER overdose

In this study, doses of SER ingested by patients with suicidal intent ranged from 450 mg to 1400 mg, with patient ages ranging from 12 to 20 years. This finding indicates that SER has become an available medication among adolescents with depression. Previous reports suggest that intentional self-harm is a common adverse event among patients under 18 years using SER (Shu et al., 2025). Among 227 adolescent suicide attempts, the most frequent method was drug overdose (69.60%), predominantly involving psychotropic medications (65.19%) (Wang et al., 2025). Commonly co-administered medications in mental health treatment include antipsychotics (olanzapine, quetiapine, risperidone, and zolpidem), mood stabilisers (magnesium valproate and lithium carbonate), antidepressants (trazodone and mirtazapine), and other agents such as benzodiazepines and probiotics. These observations highlight the need for vigilance by medical teams and relevant authorities, including careful dosage monitoring of antidepressant medications. In addition, improper storage of medications is closely associated with pediatric drug poisoning. Post-discharge medication management largely relies on parental supervision. For children with depression and a history of overdose, educating parents regarding mental illness and psychotropic drug use, and guiding appropriate storage and handling, is essential to prevent recurrence.

### 4.2 Treatment measures

SER undergoes primary metabolism in the liver, producing metabolites, including desmethyl-SER, via phase I reactions (oxidation, reduction, or hydrolysis) and phase II reactions, which conjugate metabolites with endogenous molecules. Metabolism is mediated by cytochrome P450 enzymes (mainly CYP2B6, followed by CYP2C9, 2C19, 2D6, and 3A4). Less than 0.2% of SER is excreted unchanged by the kidneys (Li et al., 2025; Chen et al., 2020). SER has a large volume of distribution, and no specific antidote exists for overdose. For acute drug poisoning, interventions such as induced vomiting and gastric lavage can effectively reduce drug absorption (Benson et al., 2013). In this study, none of the patients exhibited life-threatening symptoms or laboratory abnormalities requiring haemoperfusion or haemofiltration. Fluid replacement consisted of normal saline and oral rehydration salts, and some patients received lactulose to regulate osmotic pressure, thereby enhancing drug excretion.

### 4.3 Clinical symptoms

The most severe toxic reactions of excessive SSRIs intake affect the central nervous system, manifesting as agitation, dizziness, headache, weakness, drowsiness, insomnia, tremors, and extrapyramidal reactions (Minns and Clark, 2012). In this study, most patients exhibited neurological symptoms, likely related to excessive intake of multiple drugs. Summary of clinical manifestations: Neurological symptoms included dizziness (7 cases), drowsiness (1 case), hand tremor (3 cases), limb tremor (2 cases), unsteady gait (1 case), chest tightness (1 case), and breathing difficulty (1 case). Gastrointestinal symptoms included vomiting (12 cases), abdominal pain (3 cases), nausea (4 cases), acid reflux (1 case), and tremor of the corners of the mouth (1 case). Vomitus contained white substances (1 case) and gastric contents (1 case). General symptoms included fever (1 case; body temperature 37.4 °C), fatigue (1 case), and palpitations (2 cases). Other symptoms included nasal congestion and rhinorrhoea (1 case) and chills (possibly associated with fever). Most patients exhibited a consistent decrease in serum SER concentration, correlating with progressive amelioration of adverse reactions, consistent with previously reported concentration-dependent effects (Ahmed et al., 2008).

### 4.4 Pharmacokinetic characteristics

SER is highly protein-bound (approximately 98%), indicating a high plasma protein affinity, and has a large volume of distribution, suggesting extensive tissue penetration (Saiz-Rodriguez et al., 2018). Although conclusions cannot be drawn from a single case, pharmacokinetic characteristics in overdose appear broadly similar to those within therapeutic ranges. Owing to variability in pre-admission treatments, times to gastric lavage differed among patients, resulting in variable SER absorption and metabolism. Initial serum concentrations were 362.06 ng/mL (112.86–801.10 ng/mL), with a clearance half-life of 24.12 h (13.27–52.78 h). This result is consistent with previously reported half-lives (approximately 22–36 h) (Macqueen et al., 2001; Devane et al., 2002), although individual differences are substantial. However, overdose conditions may alter absorption and protein binding, leading to extended elimination in some patients. Furthermore, genetic polymorphisms in CYP2B6 and CYP2C19, as highlighted by an other research (Stoiljkovic et al., 2023). Depression treatment frequently involves multiple drugs with distinct mechanisms of action. Combined therapy may increase the risk of drug interactions, particularly via CYP2D6 inhibition, which can elevate SER blood concentration (Elli et al., 2025). Co-administration of benzodiazepines or atypical antipsychotic drugs may exacerbate central nervous system depression, causing drowsiness, unsteady gait, and respiratory depression. Close monitoring of adverse reactions is therefore essential during combination therapy, particularly in overdose scenarios. Critical illness markedly alters drug pharmacokinetics, including protein binding, ionization, and volume of distribution, complicating dose optimization. Polypharmacy further drives complex drug interactions, prolonging clearance and heightening treatment challenges. Thus, individualized management of SER overdose is critical. Interventions should be tailored to patient-specific metabolism, co-medication, and organ function, with timely use of activated charcoal, supportive care, and blood purification to reduce absorption and enhance elimination.

Exploratory analyses suggested associations between age, co-administration, and clearance; however, these findings should be interpreted cautiously due to the small sample size (n = 16) and lack of correction for multiple comparisons. For example, the observed effect of vomiting on half-life (β = 0.31, p = 0.04) may reflect confounding by indication rather than a true biological effect. Prospective studies with larger cohorts are warranted to validate these preliminary observations.

### 4.5 Limitations

This study has several important limitations that affect the interpretation of pharmacokinetic findings and generalizability of results.

#### 4.5.1 Critical limitations in pharmacokinetic interpretation

The interpretation of calculated half-life values (13.27–52.78 h) requires careful consideration of study design limitations that fundamentally affect pharmacokinetic validity:

Timing of Gastric Lavage Relative to Sampling: In all 16 cases, gastric lavage was performed upon hospital admission (0.67–12 h post-ingestion), and the first blood sample was collected after lavage completion (1–22 h post-ingestion). This sequence creates two critical uncertainties that preclude definitive conclusions about elimination kinetics: (a) Truncated Absorption Phase: Gastric lavage removes unabsorbed drug, potentially terminating absorption earlier than in natural overdose progression. The observed concentration-time profiles represent post-intervention kinetics rather than intrinsic overdose pharmacokinetics. Without a control group or pre-lavage samples, the impact of decontamination on apparent clearance cannot be quantified; (b) Unknown Peak Concentration Timing: The true Tmax (time to maximum concentration) could not be determined. For Patient 3, the first sample (112.86 ng/mL) was collected at 22 h post-ingestion, likely well into the elimination phase. In contrast, Patient 9’s early sample (801.1 ng/mL at 1.5 h) may approximate peak concentration, whereas Patient 16s first sample (441.27 ng/mL at 10 h) likely missed the true peak despite early admission (0.67 h). Without knowing whether the first sample preceded or followed Tmax, the calculated “half-life” may reflect mixed absorption-elimination kinetics rather than pure elimination phase behavior.

Two-Point Sampling Constraints: Half-life calculations assumed first-order elimination between two time points (C_1_, t_1_; C_2_, t_2_). However, with sampling intervals ranging from 25 to 144 h and unknown contributions from distribution phases, these estimates should be considered operational or apparent half-lives rather than definitive elimination half-lives. The distribution phase of sertraline (highly lipophilic, 98% protein-bound) may extend several hours post-peak, potentially affecting early samples. Furthermore, the assumption of first-order kinetics may not hold in overdose conditions where metabolic saturation could occur.

Therefore, the pharmacokinetic parameters reported in this study represent estimates under conditions of clinical intervention, not definitive characterization of sertraline overdose pharmacokinetics. The observed overlap with therapeutic half-life ranges (22–36 h) should be interpreted cautiously, as the wide inter-individual variability (13.27–52.78 h) likely reflects heterogeneity in absorption timing, lavage efficacy, and sampling schedules rather than true differences in elimination clearance.

#### 4.5.2 Study design and sample size constraints

The retrospective design inherently limited data completeness and standardization. The small sample size (n = 16) precludes robust statistical inference; exploratory analyses of associations between age, co-medication, vomiting, and clearance should be considered hypothesis-generating rather than confirmatory. The single-center design may limit generalizability to other populations or clinical settings.

#### 4.5.3 Absence of metabolite monitoring

A key limitation of this study is the absence of DSER measurements, which account for approximately 5%–10% of total SER activity and are metabolised via CYP 2D6 (Doherty et al., 2007). Without metabolite data, the extent of CYP450 enzyme saturation in overdose cannot be fully evaluated, which may explain the unexpectedly short half-life observed in some patients (as low as 13.27 h). DSER accumulation could theoretically prolong elimination, and future studies should employ tandem mass spectrometry to quantify both parent drug and metabolite.

In summary, while this study provides preliminary data on post-gastric lavage sertraline concentrations in adolescent overdose, the methodological limitations, particularly the inability to determine true peak concentration timing and the reliance on two-point sampling after decontamination, preclude definitive conclusions about elimination kinetics in overdose settings. These findings should be considered exploratory and require validation in prospective studies with comprehensive sampling protocols.

## 5 Conclusion

This retrospective study reports serum sertraline concentrations in 16 adolescent overdose cases managed with gastric lavage. Apparent elimination half-lives, estimated from two-point sampling post-decontamination, ranged from 13.27 to 52.78 h (median: 24.12 h). These estimates overlap with therapeutic range values but must be interpreted cautiously due to methodological limitations: unknown peak concentration timing, truncated absorption phases from gastric lavage, and inability to distinguish distribution from elimination kinetics with two-point sampling.

Exploratory analyses suggested potential associations between younger age, CYP 2D6 inhibitor co-administration, and prolonged apparent half-life, but these observations require validation in larger studies. The small sample size and retrospective design preclude definitive conclusions regarding altered elimination mechanisms in overdose.

## Data Availability

The original contributions presented in the study are included in the article/supplementary material, further inquiries can be directed to the corresponding authors.
